# Molecular Characterization and Expression Profile Analysis of Heat Shock Transcription Factors in Mungbean

**DOI:** 10.3389/fgene.2018.00736

**Published:** 2019-01-11

**Authors:** Shuai Li, Runhao Wang, Hanqi Jin, Yanhua Ding, Chunmei Cai

**Affiliations:** Key Lab of Plant Biotechnology in Universities of Shandong Province, College of Life Sciences, Qingdao Agricultural University, Qingdao, China

**Keywords:** mungbean, heat shock transcription factor, gene family, abiotic stress, gene expression

## Abstract

Heat shock transcription factors (Hsfs) are essential elements in plant signal transduction pathways that mediate gene expression in response to various abiotic stresses. Mungbean (*Vigna radiata*) is an important crop worldwide. The emergence of a genome database now allows for functional analysis of mungbean genes. In this study, we dissect the mungbean *Hsfs* using genome-wide identification and expression profiles. We characterized a total of 24 *VrHsf* genes and classified them into three groups (A, B, and C) based on their phylogeny and conserved domain structures. All *VrHsf* genes exhibit highly conserved exon-intron organization, with two exons and one intron. In addition, all VrHsf proteins contain 16 distinct motifs. Chromosome location analysis revealed that *VrHsf* genes are located on 8 of the 11 mungbean chromosomes, and that seven duplicated gene pairs had formed among them. Moreover, transcription patterns of *VrHsf* genes varied in different tissues, indicating their different roles in plant growth and development. We identified multiple stress related *cis*-elements in *VrHsf* promoter regions 2 kb upstream of the translation initiation codons, and the expression of most *VrHsf* genes was altered under different stress conditions, suggesting their potential functions in stress resistance pathways. These molecular characterization and expression profile analyses of *VrHsf* genes provide essential information for further function investigation.

## Introduction

Plants often suffer from various abiotic stresses throughout their life cycles. Therefore, they have evolved complex defense mechanisms, such as morphological modulation and transcriptome adjustment, to protect themselves from adverse conditions (Blande et al., 2014; Zeng et al., 2016; Abdelrahman et al., 2018; Zandalinas et al., 2018). Likewise, we can alter gene expression to improve plant tolerance to different stress conditions (von Koskull Döring et al., 2007; Qu et al., 2013; Li S. et al., 2016; Ohama et al., 2017). Recently, numerous regulatory components in plants, such as transcription factors, have been identified to participate in multiple signal transduction pathways in response to various environmental stresses. Under unfavorable conditions, transcription factors are regulated to activate or suppress downstream target genes to sustain plant survival (Mittler et al., 2012; Xu et al., 2017; Zupin et al., 2017; Abdelrahman et al., 2018).

Heat shock transcription factors (Hsfs) are important regulatory elements in plants that play critical roles in signal transduction to mediate gene expression in response to multiple abiotic stresses, including cold, drought, salt, and heat stresses (Collins et al., 1995; Nover et al., 2001; Guo et al., 2016; Jacob et al., 2017). *Hsf* genes regulate the transcription of the molecular chaperones, heat shock proteins (Hsps), by recognizing heat shock elements (HSEs) within their promoter regions. Activation of Hsps protects cells from impairment under stress conditions (Akerfelt et al., 2010; Scharf et al., 2012; Guo et al., 2016). *Hsf* gene activity can be regulated by different conditions. Hsf proteins form cytoplasmic complexes with Hsp90/Hsp70 chaperones to maintain their inactive states under non-stress conditions, whereas under stress conditions, Hsf proteins are released and modified, allowing them to bind their target genes (Nover et al., 2001; Akerfelt et al., 2010; Scharf et al., 2012; Westerheide et al., 2012).

Recently, many *Hsf* gene families have been identified and analyzed in more than 20 plant species at a genome-wide scale (Nover et al., 2001; Fujimoto and Nakai, 2010; Lin et al., 2011; Giorno et al., 2012; Scharf et al., 2012; Guo et al., 2016; Wei et al., 2016). For example, there are 21 *Hsf* genes in Arabidopsis, 25 in rice, 24 in tomato, 52 in soybean, 40 in cotton, and 27 in potato (Li et al., 2014; Wang et al., 2014; Guo et al., 2016; Tang et al., 2016). Similar to other transcription factor families, plant Hsf proteins share a well conserved modular structure, such as a DNA binding domain (DBD) and hydrophobic heptad repeats (HR-A/B) (Baniwal et al., 2004). The Hsf N-terminal DBD is characterized by a conserved helix-turn-helix motif, containing one 3-helical bundle (α1, α2, and α3) and one 4-stranded β-sheet (β1, β2, β3, and β4). The DBD allows Hsf proteins to recognize HSEs in target promoter regions to regulate the downstream genes (Harrison et al., 1994; Scharf et al., 2012; Guo et al., 2016). The HR-A/B, which is also known as the oligomerization domain (OD), is connected to the DBD by a flexible peptide chain. This HR-A/B domain enables Hsf proteins to form homologous trimmers to efficiently bind Hsp promoters (Scharf et al., 2012; Guo et al., 2016). Moreover, some Hsf protein functional domains also consist of nuclear localization signal (NLS), nuclear export signal (NES), and transcriptional activation (AHA) motifs. The NLS motif, which is formed by a cluster of lysine and arginine residues, and the NES, which contains many leucine residues, are close to the C-terminus in some sub-classes of Hsf proteins. In the AHA motif, the “A,” “H,” and “A” represent different kinds of amino acids. Specifically, the first “A” indicates W, F, or Y, “H” indicates L, I, V, or M, and the last “A” represents D or E (Scharf et al., 2012; Liu et al., 2016; Guo et al., 2016; Jacob et al., 2017). Based on the characteristics of their flexible peptide chain and HR-A/B regions, Hsf proteins are generally classified into three groups, HsfA, HsfB, and HsfC (Scharf et al., 2012; Guo et al., 2016).

Mungbean is one of the most important crops and is commonly consumed by humans in many countries (Tang et al., 2014; Ganesan and Xu, 2017). Mungbean seeds and sprouts contain bioactive food compounds and abundant nutrients, and have potential health benefits for humans (Tang et al., 2014; Ganesan and Xu, 2017). However, sustainable mungbean production is challenged by various stresses, such as high temperature, cold, drought etc. (Blande et al., 2014; Fragkostefanakis et al., 2015). Therefore, it is very important to characterize mungbean stress resistant genes to allow for their modification for enhanced crop adaptability. In the current study, we report the molecular identification and characterization of *VrHsf* genes in mungbean, using a combination of approaches, including sequence alignment, evolutionary relationship investigation, gene duplication and motif analysis. Moreover, we investigate the expression patterns of *VrHsf* genes in various mungbean tissues, as well as their expression profiles under different stress treatments. Our findings provide a foundation for an improved understanding of the *VrHsf* gene family in mungbean, and will be useful for further characterization of *VrHsf* gene function.

## Materials and Methods

### Plant Materials and Growth Conditions

The sequenced mungbean genotype *VC1973A* (named Zhonglu in China) was used for all experiments (Kang et al., 2014). Mungbean seeds were germinated and grown in pots in a growth room, with 16 h 28°C light/8 h 22°C dark cycles. Humidity was maintained at approximately 30%.

Two weeks old mungbean seedlings were used for stress treatments. Cold stress treatment was performed by transferring plants to a 4°C chamber for 6 h. Heat stress was carried out by putting plants in a 40°C chamber for 6 h. For the drought stress treatment, plants were grown without watering for 6 days. For the salt stress treatment, plants were watered with 200 mmol salt solution, and tissues were collected 48 h after treatment. Shoots and roots were collected separately after stress treatments and then stored at -80°C before RNA extraction. Moreover, various tissues, such as roots, stems, leaves, flowers, pods and seeds, were also sampled for gene expression analysis. Roots, stems and leaves were collected from 3 weeks old seedlings, and flowers were sampled from 6 weeks old plants. The pods were sampled for analysis at the beginning of the pod stage. The seeds, at the full seed stage were used for gene expression analysis. Each sample was analyzed using three biological replicates.

### Identification of *VrHsf* Members

The conserved amino acid sequence of DNA-binding domains (DBD, PF00447) was download from the protein family database Pfam^1^, and full-length amino acid sequences of Hsf proteins from Arabidopsis, soybean and potato were used as BLAST queries against the mungbean database^2^ and National Center for Biotechnology Information (NCBI). All output genes with default (Limit Expect Value 1e-5) were analyzed using the Pfam database (*E* = 1.0) and SMART^3^ to remove genes without conserved domain sequences. The classification of VrHsf proteins was performed using Heatster^4^. The theoretical iso-electric points, grand average of hydropathicity and protein molecular weight analyses were performed using the ProtParam tool^5^.

### Phylogenetic Relationship Analysis

In total, 124 Hsf amino acid sequences from mungbean, soybean, Arabidopsis and potato were used for phylogenetic analysis (Nover et al., 2001; Chung et al., 2013; Tang et al., 2016). The N-proximal regions of Hsf proteins, from the start of the conserved DBD domain to the end of the HR-A/B region, were aligned using Clustal-X2. The alignment result was used to construct a phylogenetic tree using MEGA 6.0’s Neighbor-Joining method with pairwise deletion, 1000 bootstraps and a Poisson model (Tamura et al., 2013).

### Analyses of *VrHsf* Gene Structures, Protein Domains, Conserved Motifs, and *cis*-Elements

The CDS and genomic DNA sequences of mungbean *Hsf* genes were aligned with the Gene Structure Display Server program (GSDS)^6^ to illustrate exon/intron organization (Hu et al., 2015). VrHsf conserved domains were obtained using Heatster^7^, Pfam and SMART. The conserved motifs of VrHsf proteins were assessed via MEME tools^8^, and the parameters of the maximum number of motifs and the optimum motif widths were 16 and 6–50 amino acid residues, respectively. Promoter *cis*-elements were analyzed using the Plant *cis*-acting regulatory DNA elements (PLACE) database (Higo et al., 1999) and the distribution maps were constructed using iBS^9^.

### Chromosomal Distribution and Duplication Analysis of *VrHsf* Genes

The MapInspect software^10^ was used for mapping *VrHsf* genes to mungbean chromosomes. Duplications of *Hsf* genes in mungbean were analyzed and marked as previously described (Zhang et al., 2015; Tang et al., 2016). The divergence time (T) of the duplicated genes was calculated as described (Lynch and Conery, 2000; Chen et al., 2014).

### RNA Extraction and Quantitative Real-Time PCR Analysis

All mungbean RNA was extracted using Qiagen RNeasy mini kit following the instructions (Qiagen, United States)^11^. cDNA synthesis was conducted as previously described (Li et al., 2017). SuperScript II reverse transcriptase first-strand synthesis kit (Invitrogen) was used for the synthesis of the first strand cDNAs with 2 μg total RNA according to the instructions. The LightCycler 480 SYBR Green I Master Kit (Roche Diagnostics) was used for quantitative real-time PCR (qRT-PCR) using a LightCycler480 machine (Roche Diagnostics), according to the manufacturer’s instructions. The amplification program for qRT-PCR was performed as previously described (Li et al., 2017). For gene expression analysis, three biological replicates were used for each sample and gene expression was normalized to an *Actin*-expressing gene in mungbean (*Vradi03g00210*). All primers used for qRT-PCR analyses are listed in Supplementary Table 1.

### Statistical Analysis

Statistical significance was analyzed by *t*-test using the SAS program (SAS Institute Inc.)^12^.

## Results

### Identification of *VrHsf* Genes in Mungbean

We used the conserved DBD amino acid sequence (PF00447) and the full-length amino acid sequences of Hsf proteins from Arabidopsis, soybean and potato as BLAST queries against the mungbean genome database and NCBI GenBank resources (Kang et al., 2014). We ultimately identified 24 *VrHsf* candidate genes in the mungbean genome with complete DBD and HR-A/B regions (Supplementary Figures 1, 2) and analyzed their genomic length, CDS length, number of amino acids, theoretical molecular weight, grand average of hydropathicity, and isoelectric point (Table 1). The genomic lengths of the *VrHsf* genes ranged from 1,435 bp (*Vradi06g15090*) to 12,757 bp (*Vradi08g09150*), the coding sequence sizes varied from 561 bp (*Vradi08g02500*) to 1,521 bp (*Vradi07g03150*), and the deduced number of amino acids ranged from 186 to 506. The grand averages of hydropathicity fell between -0.484 and -0.891, indicating that all the VrHsfs were predicted to be hydrophilic proteins. In addition, the predicted molecular weights ranged from 22.16 to 56.26 kDa, and the isoelectric points ranged from 4.69 to 9.30, indicating the structural diversity and functional variation of VrHsfs (Table 1).

**Table 1:** Identified VrHsf members.

Gene name	Gene ID	Accession number in NCBI	Chr	Genomic length (bp)	CDS length (bp)	No. of AA	Mol.Wt (kDa)	pI	GRAVY
VrHsfA1c	Vradi11g08720	XP_014521025.1	11	4045	1473	490	54.19	4.94	-0.638
VrHsfA1d	Vradi07g03150	XP_014510442.1	7	5312	1521	506	56.02	5.13	-0.656
VrHsfA1e	Vradi11g01010	XP_014521170.1	11	3448	1398	465	51.74	5.45	-0.552
VrHsfA3a	Vradi08g06500	XP_014511066.1	8	3171	1359	452	51.02	4.94	-0.648
VrHsfA3b	Vradi03g04270	XP_014495274.1	3	2686	1500	499	56.26	6.45	-0.507
VrHsfA4	Vradi10g09840	XP_022642491.1	10	3601	1161	386	43.83	5.06	-0.722
VrHsfA5a	Vradi10g02130	XP_014517147.1	10	4293	1443	480	54.04	5.60	-0.722
VrHsfA5b	Vradi0246s00340	XP_014521699.1	N/A	6346	1368	455	51.08	5.21	-0.595
VrHsfA5c	Vradi0161s00040	XP_014523429.1	N/A	2801	1209	402	45.59	5.11	-0.723
VrHsfA6a	Vradi08g00250	XP_014512857.1	8	1903	1023	340	39.45	4.69	-0.843
VrHsfA6b	Vradi08g19520	XP_014512523.1	8	2203	1035	344	39.48	5.67	-0.592
VrHsfA7a	Vradi03g01100	XP_014496397.1	3	2948	1113	370	42.68	5.32	-0.891
VrHsfA7b	Vradi08g09150	XP_022640423.1	8	12757	1077	358	41.49	5.76	-0.692
VrHsfB1	Vradi07g10520	XP_014506090.1	7	3087	762	253	28.03	6.46	-0.699
VrHsfB2a	Vradi01g14650	XP_014499538.1	1	1573	1008	335	36.38	5.51	-0.529
VrHsfB2b	Vradi11g02310	XP_014521482.1	11	2387	1065	354	38.92	5.00	-0.569
VrHsfB2c	Vradi08g19000	XP_014512458.1	8	1466	939	312	35.04	6.68	-0.569
VrHsfB3a	Vradi08g02500	XP_014511403.1	8	3255	561	186	22.16	9.30	-0.768
VrHsfB3b	Vradi03g02400	XP_014495494.1	3	2114	684	227	26.19	9.17	-0.858
VrHsfB4a	Vradi06g15090	XP_014503529.1	6	1435	1017	338	38.82	7.29	-0.641
VrHsfB4c	Vradi05g12640	XP_014499941.1	5	1669	1095	364	40.90	8.64	-0.597
VrHsfB4h	Vradi06g02880	XP_014504175.1	6	1773	819	272	31.52	6.86	-0.665
VrHsfB5	Vradi07g16260	XP_014506614.1	7	2461	654	217	25.10	6.25	-0.818
VrHsfC1	Vradi01g04120	XP_014506180.1	1	1754	942	313	35.15	6.27	-0.484


*Chr, chromosome numbers; AA, amino acid; Mol.Wt, molecular weight; pI, isoelectric point; GRAVY, grand average of hydropathicity; N/A, not applicable.*

### Classification and Phylogenetic Analysis of *VrHsf* Genes

**FIGURE 1:**
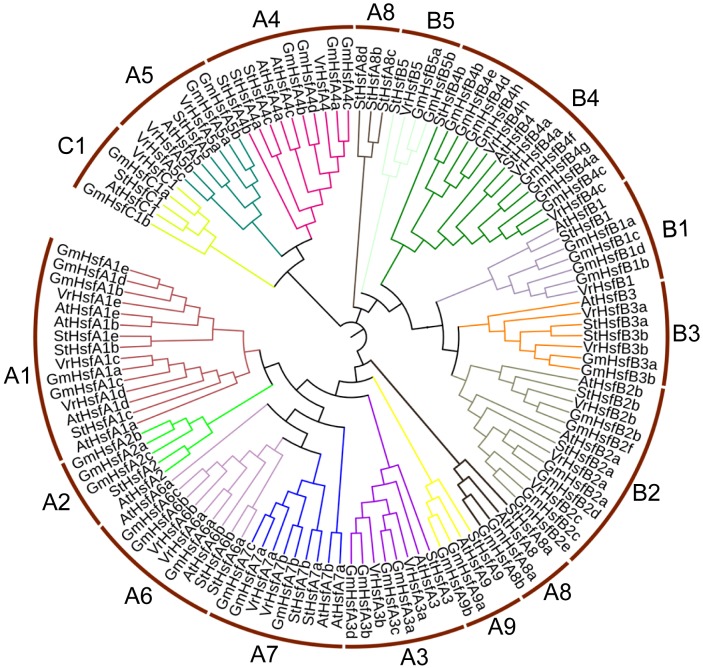
Evolutionary relationship analysis of VrHsf proteins. The N-proximal regions (from the start of the conserved DBD domain to the end of the HR-A/B region) of Hsf proteins from mungbean, soybean, Arabidopsis and potato were used to construct the phylogenetic tree using MEGA 6.0 with the Neighbor-Joining method.

**FIGURE 2:**
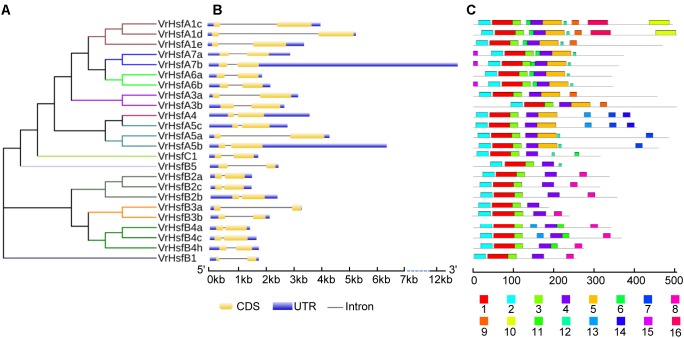
Phylogenetic and structure analysis of *VrHsf* genes. **(A)** The conserved domains of VrHsf proteins were used to construct the phylogenetic tree. **(B)** The exon/intron distribution of *VrHsf* genes were analyzed by comparing coding sequences with genomic sequences. The UTRs, exons and introns are represented by blue, yellow and black lines, respectively. **(C)** Motifs in VrHsf proteins identified by MEME. The motifs, numbered 1–16, are exhibited in different colored boxes.

The VrHsf proteins are classified into three groups (A, B, and C) based on differences in the numbers of amino acids inserted between the HR-A and HR-B cores (Guo et al., 2016). Class A has a 21 amino acid insertion between the HR-A and HR-B regions, and Class C has a 7 amino acid insertion, whereas Class B Hsfs are more compact (Scharf et al., 2012). We aligned HR-A/B regions and used Heatster to classify *VrHsfs* into three groups. Among the 24 VrHsf members, 13 VrHsf proteins were grouped into class A, 10 VrHsf members belonged to class B and only 1 member was classified as class C (Table 1 and Supplementary Figure 2). We constructed a phylogenetic tree based on 124 Hsf amino acid sequences, including 24 mungbean Hsfs, 52 soybean Hsfs (Scharf et al., 2012), 21 Arabidopsis Hsfs (Scharf et al., 2012), and 27 potato Hsfs (Tang et al., 2016), using the conserved regions from the start of the conserved DBD domain to the end of the HR-A/B domain (Figure 1 and Supplementary Table 2) to investigate the evolutionary relationships among Hsf families and to gain insight into the potential function of VrHsfs from the well-studied Hsf families in other species. VrHsfs in class A were grouped into 6 distinct sub-classes (A1, A3–A7), and no VrHsf members were classified as A2, A8, or A9. VrHsfs in class B were sub-classified into five groups (B1-B5) (Table 1, Figure 1, and Supplementary Table 2). We also created a phylogenetic tree using only VrHsf N-proximal regions (Figure 2A). The class A members formed a single group in comparison with class B, which was divided into 3 sub-groups. VrHsfB1 sub-subclass had a distinct relationship with other VrHsfB sub-families, and VrHsfB5 had a much closer relationship with class A and class C families (Figure 2A).

### Exon-Intron Organization and Conserved Motifs of *VrHsf* Genes

**FIGURE 3:**
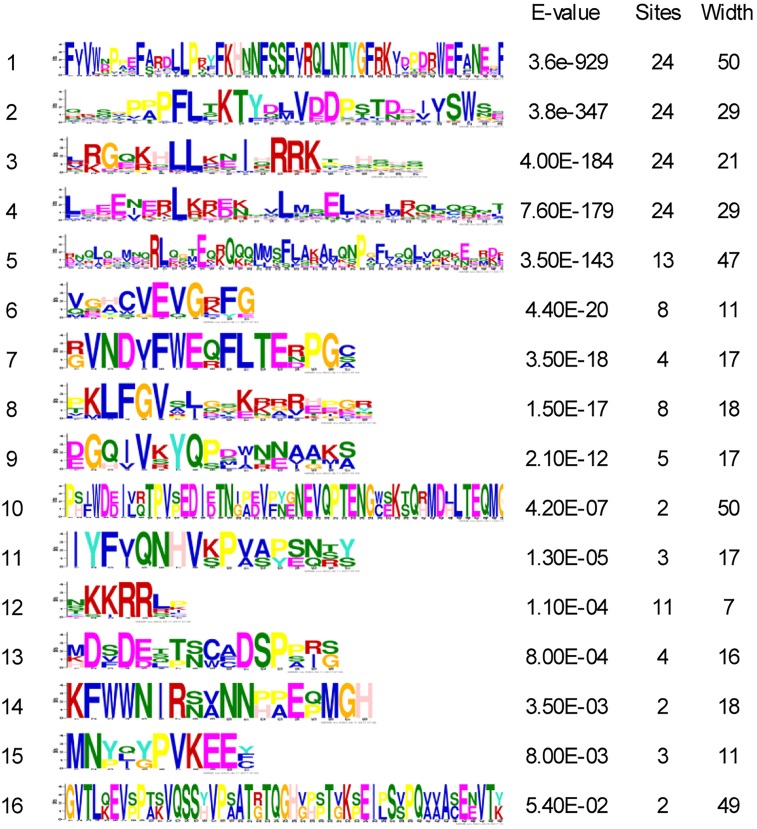
Sequence logos of 16 motifs in VrHsf proteins. The “sites” indicate the number of VrHsf proteins containing each motif. The “width” indicates the amino acid number of each motif.

We constructed the exon-intron organization of the 24 *VrHsf* genes using the genomic and coding sequences. The gene structures showed that all the *VrHsf* genes exhibited a highly conserved exon-intron organization, with two exons and one intron (Figure 2B). The intron lengths varied, similar to Arabidopsis (Nover et al., 2001). We used MEME to predict putative motifs to further reveal the conservation and diversity of *VrHsf* genes (Figures 2C, 3 and Table 2), and we identified 16 distinct motifs among mungbean Hsf proteins (Figure 3). All the *VrHsf* members contain motifs 1, 2, 3, and 4. Motif 1 is closely connected with motifs 2 and 3. Combined, motifs 1, 2, and 3 represent the most conservative domain, the DBD domain. We considered motifs 4 and 5 the HR-A/B regions (Figure 2C). Some motifs were only found in specific VrHsf proteins, for example, motif 5 is specific to the class A family, and most class B members have motif 8, except for *VrHsfB5* and *VrHsfB3a* (Figure 2C). Moreover, we identified NLS motifs in 21 VrHsf proteins and NES motifs in 9 class A and 2 class B VrHsfs. The class A Hsfs have short activator peptide motifs (AHA) and class B Hsfs contains the repressor domain tetrapeptide (Scharf et al., 2012). We detected AHA motifs, which may work as transcriptional activators, in 10 class A VrHsfs, while *VrHsfA1c, VrHsfA1d*, and *VrHsfA3b* have no AHA domains (Table 2). Eight of the 10 class B *VrHsf* genes contain the tetrapeptide LFGV, the RD domain (Table 2), and therefore may function as repressors in stress response pathways (Scharf et al., 2012). In addition, *VrHsfC1* has no RD or AHA domains. In sum, these observations indicate the functional divergence among *VrHsf* genes.

**Table 2:** Functional domains of mungbean VrHsf proteins.

Gene name	DBD	HR-A/B	RD	NLS	AHA	NES
VrHsfA1c	18–111	135–199	nd	(217)RRISEVNKKRR	nd	(476)LTEQMGLL
VrHsfA1d	25–118	142–206	nd	(224)RRISEANKKRR	nd	(492)LTEQMGLL
VrHsfA1e	12–105	127–191	nd	(209)KHITGANKKRR	(400)DEFWELFFRP	(454)LTKQMGLL
VrHsfA3a	20–113	145–191	nd	(217)RVVRKFVKQH	(367)LEDIWDS;(386)NELWGN; (405)SDMSESDIWD; (422)IDKWPGD	nd
VrHsfA3b	97–190	219–265	nd	(291)KVRRRFVKQH	nd	nd
VrHsfA4	12–105	131–188	nd	(206)RKRR	(257)IMFWENIAHD; (336)DIFWERFLTE	nd
VrHsfA5a	16–109	129–186	nd	(197)RKIESMDLSAYKKRRL	(428)DVFWEQFLTE	(468)ISRNIKNM
VrHsfA5b	19–112	158–188	nd	(199)RKIESMDLLAYNKKRR	(402)DVFWEQFLTE	(442)RISGNVMD
VrHsfA5c	11–104	128–185	nd	(203)RKRR	(254)VAFWEAIAHD; (338)DVFWEQFLTE	(389)HAEPMGHV
VrHsfA6a	34–127	143–207	nd	(222)WRKELEEAISSKRRR	(305)EVLWEELLNE	nd
VrHsfA6b	42–135	151–215	nd	(230)LRKELKEAFSKKRRS	(310)EVFWQDLLNE	(329)VDVLARQLGYL
VrHsfA7a	49–142	158–222	nd	(237)KRKELEEAMSKKRRR	(326)EGFWEELFSE	(355)VNVLANRFGYL
VrHsfA7b	42–135	160–210	nd	Nd	(314)DEEFWEELM	(275)VSELEKLAMEM
VrHsfB1	7–100	144–181	(230)KLFGVWL	Nd	nd	nd
VrHsfB2a	28–121	177–213	(263)KLFGVAI	(272)KRARE	nd	nd
VrHsfB2b	23–116	184–220	(283)KLFGVSI	(292)KRCRT	nd	nd
VrHsfB2c	21–114	170–206	(240)KLFGVAI	(249)KRARE	nd	nd
VrHsfB3a	18–111	134–169	nd	Nd	nd	nd
VrHsfB3b	20–113	144–179	(193)MLFGVRL	(210)NR	nd	nd
VrHsfB4a	22–115	177–213	(289)KLFGVSL	(299)KRVH	nd	(320)LLVENDDFFGLNLM
VrHsfB4c	22–115	190–226	(320)KLFGVSL	(330)KRVH	nd	(347)LVLEKDDLGLNLM
VrHsfB4h	23–116	162–198	(251)KLFGVPL	(261)KRLH	nd	nd
VrHsfB5	47–143	170–210	nd	(212)KIRR	nd	nd
VrHsfC1	14–108	131–174	nd	(194)EKKRRL	nd	nd


*DBD, DNA-binding domain; HR-A/B, heptad pattern of hydrophobic amino acid residues; RD, tetrapeptid motif LFGV as core of repressor domain; NLS, nuclear localization signal; AHA, Aromatic (W, F, Y), large Hydrophobic (L, I, V), and Acidic (E, D) amino acid residues; NES, Nuclear export signal. Numbers in DBD and HR-A/B columns indicate positions of the first and the end amino acid of the these domains, and numbers in brackets mean the position of the first amino acid of RD, NLS, AHA, and NES domains, respectively. nd, no domains detected.*

### Chromosomal Location and Duplication Analysis of *VrHsf* Genes

**FIGURE 4:**
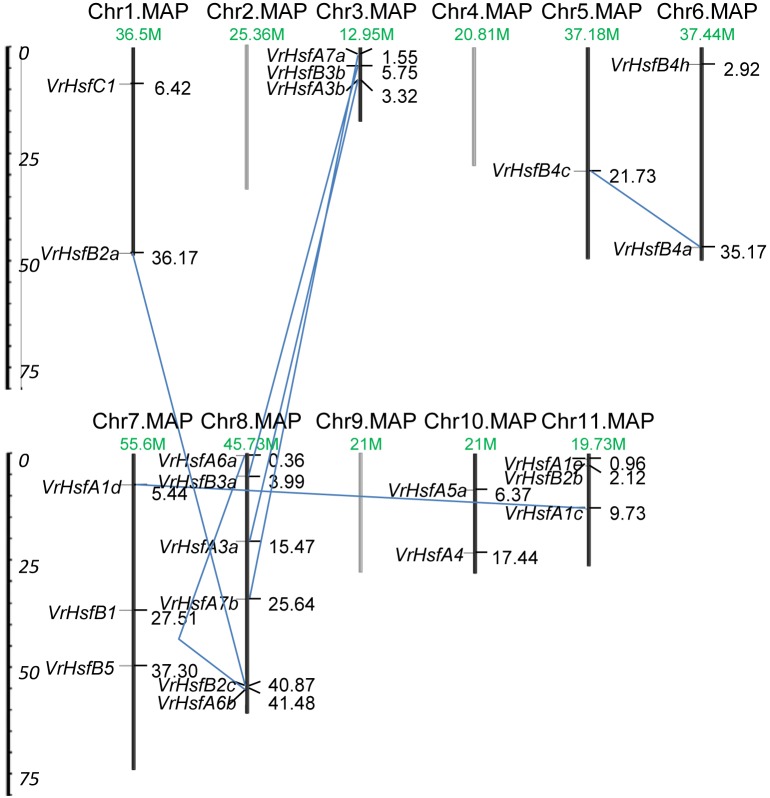
Chromosomal location and duplication analysis of *VrHsf* genes. Chromosome number and length are represented. The positions for each *VrHsf* gene are shown on the chromosome. The blue lines indicate segmental duplications.

The *VrHsf* gene family members have some common functions, and some members may have evolved new functions related to the original genes after gene duplication. The chromosomal locations of *VrHsf* genes can represent gene distributions after duplication. To map the locations of *VrHsf* genes on different chromosomes, we determined the distribution of *VrHsf* genes based on the mungbean genome database. Two candidate genes, *VrHsfA5b* and *VrHsfA5c*, were discarded due to the lack of chromosome information (Table 1). *VrHsf* genes were located on 8 of the 11 mungbean chromosomes, with no *VrHsfs* found on chromosomes 2, 4, or 9 (Figure 4). Chromosome 8 contained the most *VrHsf* genes, with four class A and two class B genes (*VrHsfA3a, VrHsfA6a, VrHsfA6b, VrHsfA7b, VrHsfB2c*, and *VrHsfB3a*), followed by chromosomes 3, 7, and 11, with three genes each (Figure 4). To investigate the duplication events, which may have occurred during mungbean genome evolution, we used the MpInspect software to analyze mungbean *Hsf* genes. The analysis identified six interchromosomal duplications and one intrachromosomal duplication (Figure 4). Class A proteins contained four duplication events, class B contained three duplication events and class C had no duplicated genes, and these gene duplications indicated similar functions for the duplicated gene pairs. In addition, chromosome 8 contained the most duplicated gene pairs, with five duplication events (*VrHsfB2a/VrHsfB2c, VrHsfB3a/VrHsfB3b, VrHsfA3a/VrHsfA3b, VrHsfA6a/VrHsfA6b*, and *VrHsfA7a/VrHsfA7b*) and all *VrHsf* members located on chromosome 8 had duplicated genes, indicating that chromosome 8 contained the original genes of many duplicated *VrHsf* genes. In contrast, genes on chromosome 10 had no duplication events (Figure 4).

### Promoter Structures of *VrHsf* Genes

**FIGURE 5:**
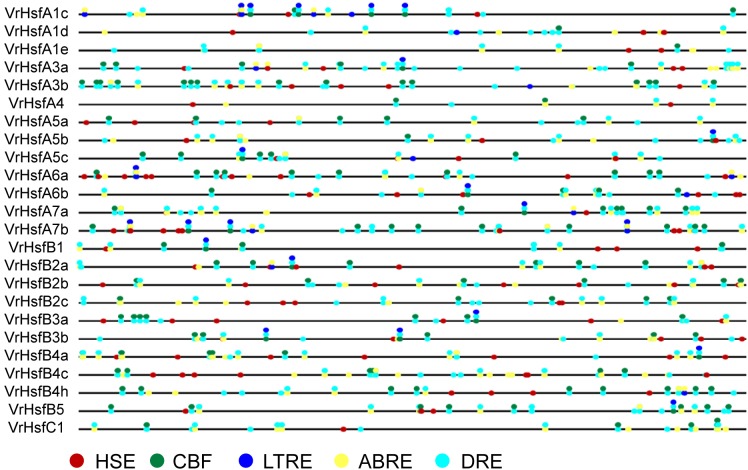
*Cis*-element analysis of *VrHsf* promoters 2 kb upstream of the translation initiation codons. Different colored circles indicate different response elements. HSE, Heat Stress Element; CBF, C-Repeat Binding Factor; ABRE, ABA Responsive Element; DRE, Dehydration-Responsive Element; LTRE, Low Temperature Responsive Element.

We next investigated putative *cis*-elements in the promoter region 2 kb upstream of the translation initiation codons of *VrHsf* members. We obtained many *cis*-elements in the promoter regions including five known abiotic stress response elements (Figure 5). The stress related elements, Heat Stress Element (HSE), Low Temperature Responsive Element (LTRE), Dehydration-Responsive Element (DRE), C-Repeat Binding Factor (CBF), and ABA Responsive Element (ABRE) were characterized in *VrHsf* promoter regions (Figure 5). Distribution analysis of these *cis*-elements showed that all the *VrHsf* promoters contain HSE, CBF, ABRE, and DRE elements, and 17 of the 24 *VrHsf* promoter regions contain LTRE elements. All genes had multiple DREs and ABREs, pointing to their key roles in response to drought stress and ABA response pathways (Figure 5). These observations of the *cis*-elements in promoter regions imply that the *VrHsf* genes exhibit functional diversity and might be responsive to many different abiotic stresses.

### *VrHsf* Gene Expression Analysis in Multiple Tissues

**FIGURE 6:**
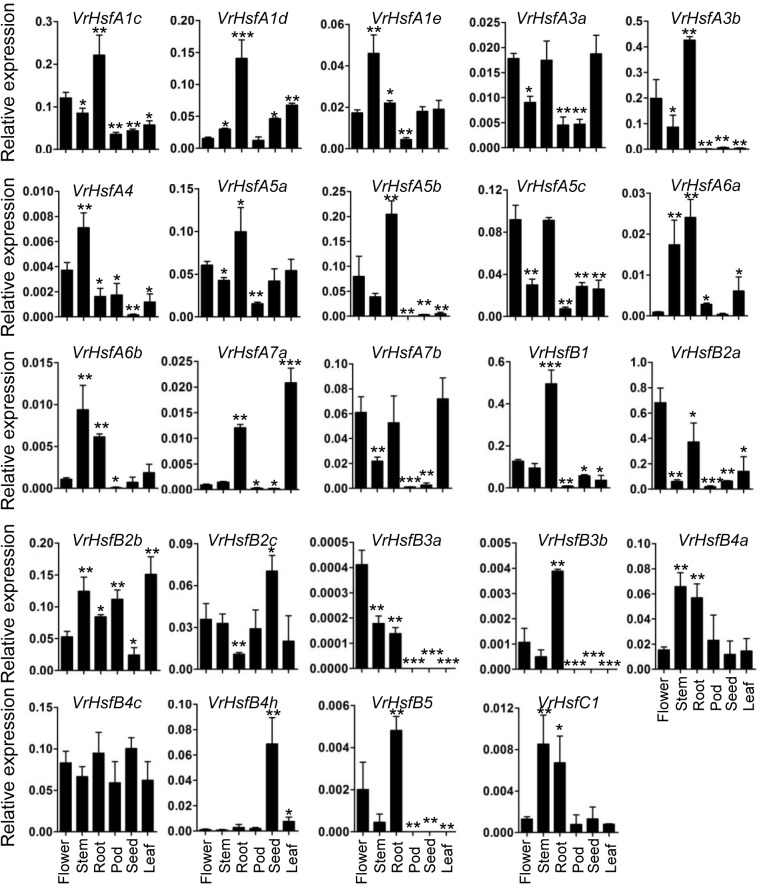
Expression analysis of *VrHsf* genes in different tissues. Tissues, such as flower, stem, root, leaf pod and seed were used for analysis. Each sample was analyzed using three biological replicates and normalized to an *Actin*-expressing gene in mungbean (*Vradi03g00210*). ^∗∗∗^, ^∗∗^, and ^∗^ are significantly different at *P* < 0.001, *P* < 0.01, and *P* < 0.05, respectively, compared with flower.

To address the potential functions of *VrHsf* genes in different tissues, we used qRT-PCR to analyze their transcription patterns in various tissues, including the root, stem, leaf, flower, pod and seed (Figure 6). The expression patterns of each *VrHsf* gene varied in different tissues, indicating their potential functions in these tissues. Among the class A family, *VrHsfA1c, VrHsfA1d*, and *VrHsfA5a* were expressed at relatively high levels in all tissues, while *VrHsfA4* was expressed at low levels (Figure 6). Moreover, *VrHsfA1c, VrHsfA1d*, and *VrHsfA5a* were more highly expressed in the root than in other tissues, indicating their critical roles in roots. Among the *VrHsfB* family, *VrHsfB2a, VrHsfB2b*, and *VrHsfB4c* were also expressed at high abundances in all tissues. In contrast, *VrHsfB3a, VrHsfB3b*, and *VrHsfB5* were expressed at low levels, and were not expressed in the pod, seed, and leaf (Figure 6). In addition, the *VrHsfC1* gene showed constitutively low expression in all tissues (Figure 6).

Many duplicated gene pairs displayed similar expression patterns in some tissues (Figure 6). For example, *VrHsfA1c* and *VrHsfA1d* showed similar expression levels in the root, leaf and seed. *VrHsfA6a* and *VrHsfA6b* expression patterns closely resembled each other in the flower and stem. *VrHsfB4a* and *VrHsfB4c* displayed similar expression patterns in the stem. Moreover, expression of *VrHsfB3a* conformed to *VrHsfB3b* in the pod, seed and leaf, indicating closely related functions in these tissues for these duplicated genes. In contrast, duplicated genes showed distinct expression levels in some tissues (Figure 6), indicating that they may have evolved new functions compared to the original genes.

### Expression Analysis of *VrHsf* Genes in Stress Responses

**FIGURE 7:**
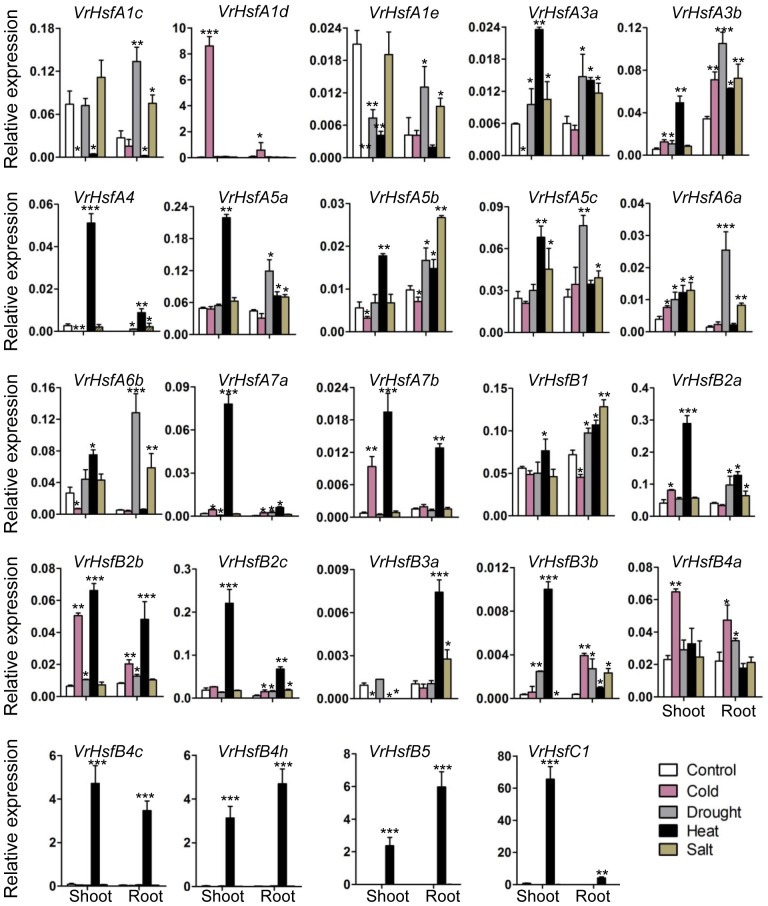
Expression analysis of *VrHsf* genes in response to different stresses. Two weeks old plants were used for various stress treatments. Each sample was analyzed using three biological replicates and normalized to an *Actin*-expressing gene in mungbean (*Vradi03g00210*). ^∗∗∗^, ^∗∗^, and ^∗^ are significantly different at *P* < 0.001, *P* < 0.01, and *P* < 0.05, respectively, compared with the relative control.

To investigate the potential functions of *VrHsf* genes in response to different stresses, we analyzed *VrHsf* gene expression in mungbean shoots and roots under cold, drought, heat and salt conditions. The expression of most *VrHsf* genes changed in roots or shoots under stress treatments (Figure 7). The expression patterns of each *VrHsf* gene varied under different stress treatments. Expression of *VrHsfA1d, VrHsfA7b, VrHsfB2b*, and *VrHsfB4a* increased sharply under cold treatment in the shoot. Under drought stress, *VrHsfA6a* and *VrHsfA6b* exhibited a more than 10-fold increase in expression level in the root, compared with plants grown under normal conditions. The expression of many genes increased more than 10-fold under heat stress, including *VrHsfA4, VrHsfA7a, VrHsfA7b, VrHsfB2c, VrHsfB4c, VrHsfB4h, VrHsfB5*, and *VrHsfC1* (Figure 7). Under salt treatment, *VrHsfA6b* showed the greatest change in expression with more than an 11-fold increase. The expression alterations of these *VrHsf* genes suggest their potential roles in response to the related stresses. In addition, the duplicated gene pairs displayed similar expression patterns under certain stresses (Figure 7), such as *VrHsfA3a*/*VrHsfA3b, VrHsfA7a*/*VrHsfA7b*, and *VrHsfB2a*/*VrHsfB2c* in both root and shoot under heat stress, *VrHsfA6a*/*VrHsfA6b* in root under drought stress, suggesting the functional redundancy of these duplicated genes.

## Discussion

During the past decades, the identification of *Hsf* genes in many species has greatly increased our knowledge of the molecular mechanisms of plant developmental and defense processes (Scharf et al., 2012; Guo et al., 2016; Wang et al., 2017). Mungbean is an important crop in the world and the emergence of its genome database allows functional analysis of mungbean genes (Kang et al., 2014). In this study, we identified 24 *VrHsf* genes and investigated their characteristics using the mungbean genome database.

Different numbers of *Hsf* genes have been found in different species. Mungbean contains 24 *VrHsfs* that are similar to Arabidopsis, tomato, rice and potato, but different from the legume plants peanut and soybean (Scharf et al., 2012; Tang et al., 2016; Wang et al., 2017). This difference is most likely due to the fact that soybean and peanut had two duplications during evolution, while only one duplication occurred in mungbean (Chung et al., 2013; Kang et al., 2014; Tang et al., 2016). Although gene numbers increased due to double duplications in a variety of species, some duplicated genes lost functions in the evolution process (Wang et al., 2015). Therefore, there may be many more non-functional *Hsf* genes in soybean and peanut than in mungbean. The sub-classes A2, A8, and A9, which have been identified in many species (Scharf et al., 2012; Wang et al., 2017), were not found in mungbean (Figure 1 and Table 1). Most of the sub-classes are shared among many species, but some clusters were lost during evolution in some plants. For example, peanut does not contain the A3, A6a, A7, B3, and B4 sub-classes (Wang et al., 2017). These observations imply the functional conservation and divergence of *Hsf* genes among different plants. Moreover, sub-classes of mungbean A family *Hsf* genes were closely clustered, compared with B family *Hsf* genes, which include three clades. And *VrHsfB5* displayed a closer relationship with A family genes (Figure 2A), indicating the functional diversity of mungbean B family *Hsf* genes. In conclusion, since many plant species grow in different conditions, the evolved diversity of *Hsf* genes may contribute significantly to the plants’ survival and adaption to the environment.

Although most of the *Hsf* genes play critical roles in response to abiotic stresses, *Hsf* genes show remarkable functional diversification in Arabidopsis, for example, *AtHsfA3* works as part of drought stress signaling and *AtHsfA9* controls *Hsp* expression during seed development (Scharf et al., 2012). *VrHsf* genes were classified into three classes based on the gene structure diversity (Figure 1), indicating their function diversity for different classes of *VrHsfs* genes. In addition, some *VrHsf* genes classified into the same sub-classes contained different conserved domains, such as *VrHsfA1c* and *VrHsfA1e* (Figure 3 and Table 2), which suggested that they might have different functions and cannot replace each other in the stress response pathway.

The involvement of *Hsf* genes in plant growth and development has been revealed in past decades, and homologous genes exhibit either functional conservation or divergence in different species (Scharf et al., 2012; Guo et al., 2016). Expression profiles of *VrHsf* genes in different tissues may be closely related to their functions in organ development. For example, the expression of *VrHsfB4h* is higher in seeds than any other tested tissues (Figure 6), indicating its potential function in seed development. However, the expression pattern of *AtHsfB4*, the homologous gene in Arabidopsis, is distinct from *VrHsfB4h* (Figure 1). Overexpression of *AtHsfB4* in Arabidopsis induces specific effects on root development (Begum et al., 2013). Although homologous genes often have similar functions in different species, the evolution of plants enables gene function diversification. *AtHsfB4* has three homologous genes in mungbean, *VrHsfB4a, VrHsfB4c*, and *VrHsfB4h* (Figure 1). In addition to the low expression of *VrHsfB4h* in root, *VrHsfB4a* and *VrHsfB4c* might have some functions in common with *AtHsfB4* in root development.

The *Hsf* genes play critical roles in protecting plants from stresses (Scharf et al., 2012; Guo et al., 2016). Most *VrHsf* genes contain multiple HSE, CBF, ABRE, DRE, and LTRE *cis*-elements (Figure 5), which suggest that *VrHsf* genes could be involved in various stress responses, such as heat and drought. Accordingly, most of the *VrHsf* gene expression patterns change under stress conditions in roots or shoots (Figure 7). The numbers and types of *cis*-elements are varied among the *VrHsf* promoters, resulting in different expression profiles of *VrHsf* genes in the tested tissues and under different stresses (Figures 6, 7), exemplifying the functional diversity of these *VrHsf* genes. However, for some *VrHsf* genes, although they contained these *cis*-elements in the promoter regions, their expression levels did not change under certain stresses. For example, *VrHsfA1c* had all five *cis*-elements in its promoter region, but did not show changes in expression in shoots under drought conditions. Moreover, the expression level of *VrHsfA3a* in roots under cold stresses is similar to that in normal conditions (Figures 5, 7). One possibility for this might be that the treatment time was not sufficient to activate *VrHsfA3a* gene expression. In addition, epigenetic and somatic genome variations also play important roles in stress responses (Li X. et al., 2016), although these *VrHsf* genes did not show expression alterations, they may have some epigenetic and somatic genome variations, which require further investigation.

Gene duplication is particularly prevalent in plants and is an essential source for evolution. The duplicated genes can be lost, pseudogenized or become novel genes (Kondrashov et al., 2002; Baskaran et al., 2017). We identified seven duplicated gene pairs in the mungbean genome (Figure 4), all of which were generated between 73.88 and 91.01 MYA (Lynch and Conery, 2000; Chen et al., 2014). However, mungbean experienced one ancient whole-genome duplication (WGD) 58 MYA (Kang et al., 2014). Thus, the duplication of *VrHsfs* occurred early before the WGD. The seven duplicated gene pairs showed either similar or divergent expression levels (Figures 6, 7). The *Hsf* duplicated genes also displayed functional divergence in plants (Zhang et al., 2016). In mungbean, some duplicated gene pairs also showed functional divergence during evolution (Figures 6, 7). For example, *VrHsfB2a* exhibited higher expression levels than its duplicated gene *VrHsfB2c* in flower and root (Figure 6). Moreover, *VrHsfB3b* showed increased expression under heat stress in shoot, while its duplicated gene *VrHsfB3a* displayed decreased transcription level under heat stress in shoot (Figure 7). And also *VrHsfB3a* and *VrHsfB3b* showed different *cis*-element array in the promoter region (Figure 5), which may be responsible for their expression alterations under stresses conditions. Transposable elements (TEs) can move genes or gene fragments to new chromosomal positions, and create new duplications (Jiang et al., 2004; Lai et al., 2005). Among the duplicated genes, *VrHsfA7b* contained an HSF-type DNA-binding domain and a plant transposase domain, belonging to the *Ptta/En/Spm* gene family (Supplementary Figure 3). Thus, *VrHsf7b* may not only have Hsf protein functions, but may also work as a transposase that may have produced its duplicated gene *VrHsf7a* (Figure 4). The transposition of the *En/Spm* family is regulated through interacting autoregulatory or epigenetic mechanisms (Fedoroff, 1999; Staginnus et al., 2001), which might explain why *VrHsf7a* and *VrHsf7b* are located on different chromosomes (Table 1).

In summary, we have characterized *Hsf* genes in mungbean, and shown that their expression profile changes in response to stresses. *VrHsf* gene modification might improve abiotic stress tolerance of mungbean plants. However, much work remains to fully understand the mechanisms of *VrHsf* gene functions in stress responses.

## Author Contributions

SL and CC conceived and designed the research. SL, RW, YD, and HJ conducted the experiments and analyzed the data. SL wrote the manuscript with the input of RW. All authors read and approved the manuscript.

## Conflict of Interest Statement

The authors declare that the research was conducted in the absence of any commercial or financial relationships that could be construed as a potential conflict of interest.

## Footnotes

1 http://pfam.xfam.org/search

2 http://plantgenomics.snu.ac.kr/mediawiki-1.21.3/index.php/Main_Page

3 http://smart.embl-heidelberg.de/

4 http://www.cibiv.at/services/hsf/

5 https://web.expasy.org/protparam/

6 http://gsds.cbi.pku.edu.cn/index.php

7 http://www.cibiv.at/services/hsf/

8 http://meme.sdsc.edu/meme/intro.html

9 http://ibs.biocuckoo.org/

10 http://www.mybiosoftware.com/mapinspect-compare-display-linkage-maps.html

11 http://www.qiagen.com

12 http://www.sas.com
