# Supplementary material for: Molecular Characterization and Expression Profile Analysis of Heat Shock Transcription Factors in Mungbean
Source: Front Genet. 2019 Jan 11;9:736. doi: 10.3389/fgene.2018.00736 (PMC6336897; doi:10.3389/fgene.2018.00736)
Supplement: Supplementary file 1 [file Data_Sheet_1.docx]

**Molecular characterization and expression profile analysis of heat shock transcription factors in mungbean**

**Running title: Identification of mungbean *Hsfs***

Shuai Li^1,#^ ^*^, Runhao Wang^1,#^, Hanqi Jin^1,#^,Yanhua Ding^1^ and Chunmei Cai^1 *^

***Correspondence:** Dr. Shuai Li: [li2014shuai@163.com](mailto:li2014shuai@163.com) or Dr. Chunmei Cai: [caichunmei0902@163.com](mailto:caichunmei0902@163.com)

**Supplementary Table 1. Primers used in this study**

| **Gene name** | **Forward primer(5′→3′)** | **Reverse primer(5′→3′)** |
| --- | --- | --- |
| *VrHsfA1c* | GCAGACTCCAGTTCCAGAGG | CAGGCCTTGGACAAAATGAT |
| *VrHsfA1e* | CCAAGCCCACTTACAGGAGA | GCTCAGCAGCAAAGGACCTA |
| *VrHsfA1d* | CACTGCCCCTTGATTTTGAT | GATACCGGAGTTCGCACAAT |
| *VrHsfA3a* | CTGGTTCAAGCTGTGGGAAT | GGCCTATCTTCTTTTGGCTGA |
| *VrHsfA4* | GTGGCTGCTGGTGTTAATGA | GACACTGATCAGCCTTGCAC |
| *VrHsfA5c* | GACAGAGGATCCTGGTGCAT | AAGTTTTCTCTGCCTGACCAA |
| *VrHsfA5a* | CGGACAGGGTGAATGATGTT | TGTCCTAGACGGCATGATGA |
| *VrHsfA5b* | GAGGCTACCCCAACTGCTC | GTGTGAGCTGATCCATGTCC |
| *VrHsfA6a* | GCTTGCTATGAACATGGAGGA | TCATTATCATCATTTTCCAATGC |
| *VrHsfA6b* | GAGCAAATTGGAAGCCAGAA | AGTTGCCGAGCCAATACATC |
| *VrHsfA7a* | GGGAAAGAGGGAACAAGAGG | TCATGATCCTATGTGGTTCATTT |
| *VrHsfA7b* | GATCCGCATACCTTCTCTGC | TGCTTATGCCCTCTGATGAA |
| *VrHsfA3b* | GGAAGTTGCGGATCTGAGAA | CGCTCTCTTGTGGTTGTTGT |
| *VrHsfB1* | GCGGTGCGTTTTGAATGTAT | TCCATGATGACCGAGAAAAA |
| *VrHsfB2a* | GTGGAACCCTACCGTCTTTG | TGAATTTCGCTCAGAAGCTG |
| *VrHsfB2b* | AGCTCAGGCACAGACACAGA | TCACGTGCCTCATGTCATTT |
| *VrHsfB2c* | GGTACAAAGCGAGCGAGAGA | GACGCCGCCTAAATTAACCT |
| *VrHsfB3b* | GAGACAAGACAACTTGCGACA | GGGTGTTGCTGGAGATTGTT |
| *VrHsfB3a* | GCGATTCAGAAAGGGAGAGA | GGTGACCAAATCGAGCAAGT |
| *VrHsfB4a* | CAACAGTAAGCAAGCGCGTA | TGAAGGGGGCATGAGATTTA |
| *VrHsfB4c* | AGTTTGCCAGGGACCTTCTT | GGTGATTCATGCTCCCTTGT |
| *VrHsfB4h* | TCCTATGAACAAAGAAGCAGCA | CAAGGTTACCTGGGTGCAAT |
| *VrHsfB5* | AAAAAGTGCGAGCCAAGTGT | TTGTTGGGTATCCATAAACTGAGA |
| *VrHsfC1* | GGGGAACAGGGATGTTATGG | ACCTTCCAACAACGAAAACG |
| *Vradi03g00210* | CAGTGTCTGGATTGGAGGCT | GTCCTCGACCACTTGATG |

**Supplementary Table 2. Plant *Hsfs* used for phylogenetic tree construction**

| Hsfs |  | Arabidopsis | Soybean | Potato | Mungbean |
| --- | --- | --- | --- | --- | --- |
| HsfA1 | a | At4g17750 | Glyma09g206600 |  |  |
|  | b | At5g16820 | Glyma11g009800 | PGSC0003DMP400005713 |  |
|  | c |  | Glyma01g015900 | PGSC0003DMP400026069 | Vradi11g08720 |
|  | d | At1g32330 | Glyma16g091800 |  | Vradi07g03150 |
|  | e | At3g02990 | Glyma01g233000 | PGSC0003DMP400046951 | Vradi11g01010 |
| HsfA2 | a | At2g26150 | Glyma14g096800 | PGSC0003DMP400014459 |  |
|  | b |  | Glyma17g227600 |  |  |
|  | c |  | Glyma04g052000 |  |  |
| HsfA3 | a | At5g03720 | Glyma10g066100 | PGSC0003DMP400004805 | Vradi08g06500 |
|  | b |  | Glyma03g191100 |  | Vradi03g04270 |
|  | c |  | Glyma13g151200 |  |  |
|  | d |  | Glyma19g37580 * |  |  |
| HsfA4 | a | At4g18880 | Glyma13g225700 | PGSC0003DMP400048368 | Vradi10g09840 |
|  | b |  | Glyma05g162300 | PGSC0003DMP400030290 |  |
|  | c | At5g45710 | Glyma15g086400 | PGSC0003DMP400049433 |  |
|  | d |  | Glyma08g119900 |  |  |
| HsfA5 | a | At4g13980 | Glyma05g151800 | PGSC0003DMP400008251 | Vradi10g02130 |
|  | b |  | Glyma08g108600 |  | Vradi0246s00340 |
|  | c |  |  |  | Vradi0161s00040 |
| HsfA6 | a | At5g43840 | Glyma10g003100 | PGSC0003DMP400011443 | Vradi08g00250 |
|  | b | At3g22830 | Glyma20g150300 | PGSC0003DMP400028424 | Vradi08g19520 |
|  | c |  | Glyma10g244000 |  |  |
| HsfA7 | a | At3g51910 | Glyma19g159500 | PGSC0003DMP400033606 | Vradi03g01100 |
|  | b | At3g63350 | Glyma10g029600 | PGSC0003DMP400033608 | Vradi08g09150 |
|  | c |  | Glyma03g31380 * |  |  |
| HsfA8 | a | At1g67970 | Glyma08g047400 | PGSC0003DMP400030540 |  |
|  | b |  | Glyma05g240500 | PGSC0003DMP400063465 |  |
|  | c |  |  | PGSC0003DMP400065338 |  |
|  | d |  |  | PGSC0003DMP400049438 |  |
| HsfA9 | a | At5g54070 | Glyma13g105700 | PGSC0003DMP400055694 |  |
|  | b |  | Glyma17g053700 |  |  |
| HsfB1 | a | At4g36990 | Glyma01g185800 | PGSC0003DMP400007176 | Vradi07g10520 |
|  | b |  | Glyma17g174900 |  |  |
|  | c |  | Glyma11g056200 |  |  |
|  | d |  | Glyma05g095900 |  |  |
| HsfB2 | a | At5g62020 | Glyma09g143200 | PGSC0003DMP400025228 | Vradi01g14650 |
|  | b | At4g11660 | Glyma11g025700 | PGSC0003DMP400005485 | Vradi11g02310 |
|  | c |  | Glyma10g237800 |  | Vradi08g19000 |
|  | d |  | Glyma16g196200 |  |  |
|  | e |  | Glyma20g156800 |  |  |
|  | f |  | Glyma01g217400 |  |  |
| HsfB3 | a | At2g41690 | Glyma19g137800 | PGSC0003DMP400047457 | Vradi08g02500 |
|  | b |  | Glyma03g135800 | PGSC0003DMP400014364 | Vradi03g02400 |
| HsfB4 | a | At1g46264 | Glyma04g039600 | PGSC0003DMP400014004 | Vradi06g15090 |
|  | b |  | Glyma20g042000 | PGSC0003DMP400056532 |  |
|  | c |  | Glyma06g040900 |  | Vradi05g12640 |
|  | d |  | Glyma14g036200 |  |  |
|  | e |  | Glyma07g235600 |  |  |
|  | f |  | Glyma14g083700 |  |  |
|  | g |  | Glyma17g241300 |  |  |
|  | h |  | Glyma02g278400 |  | Vradi06g02880 |
| HsfB5 | a |  | Glyma13g180200 | PGSC0003DMP400051767 | Vradi07g16260 |
|  | b |  | Glyma01g143500 |  |  |
| HsfC1 | a | At3g24520 | Glyma09g190600 | PGSC0003DMP400000756 | Vradi01g04120 |
|  | b |  | Glyma07g053300 |  |  |
| * means gene ID obtained from soybean genome version 1. The soybean and Arabidopsis Hsfs were obtained (Scharf et al., 2012) and potato Hsfs were obtained (Tang et al., 2016). | | | | | |
|  |  |  |  |  |  |


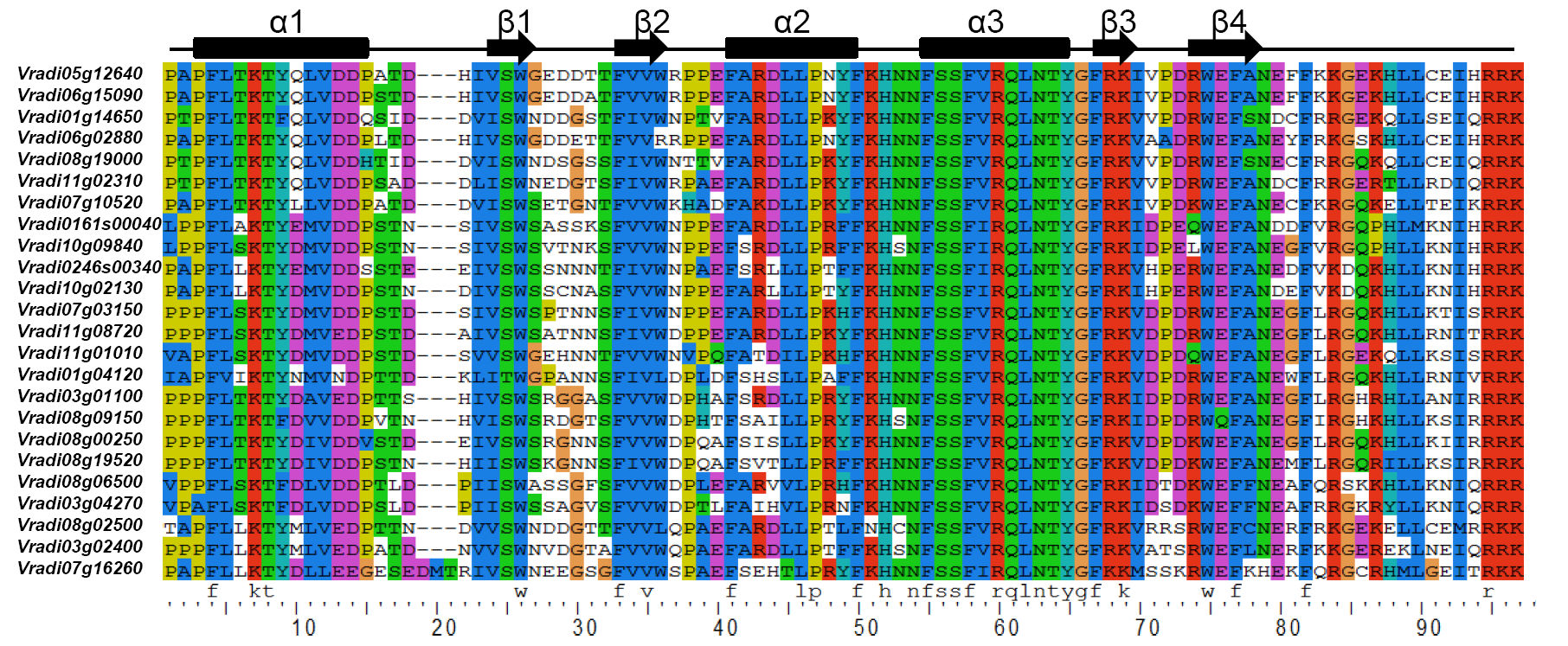


**Supplementary Figure 1. Alignment of VrHsf DNA-binding domains.** The conserved amino residues were listed.


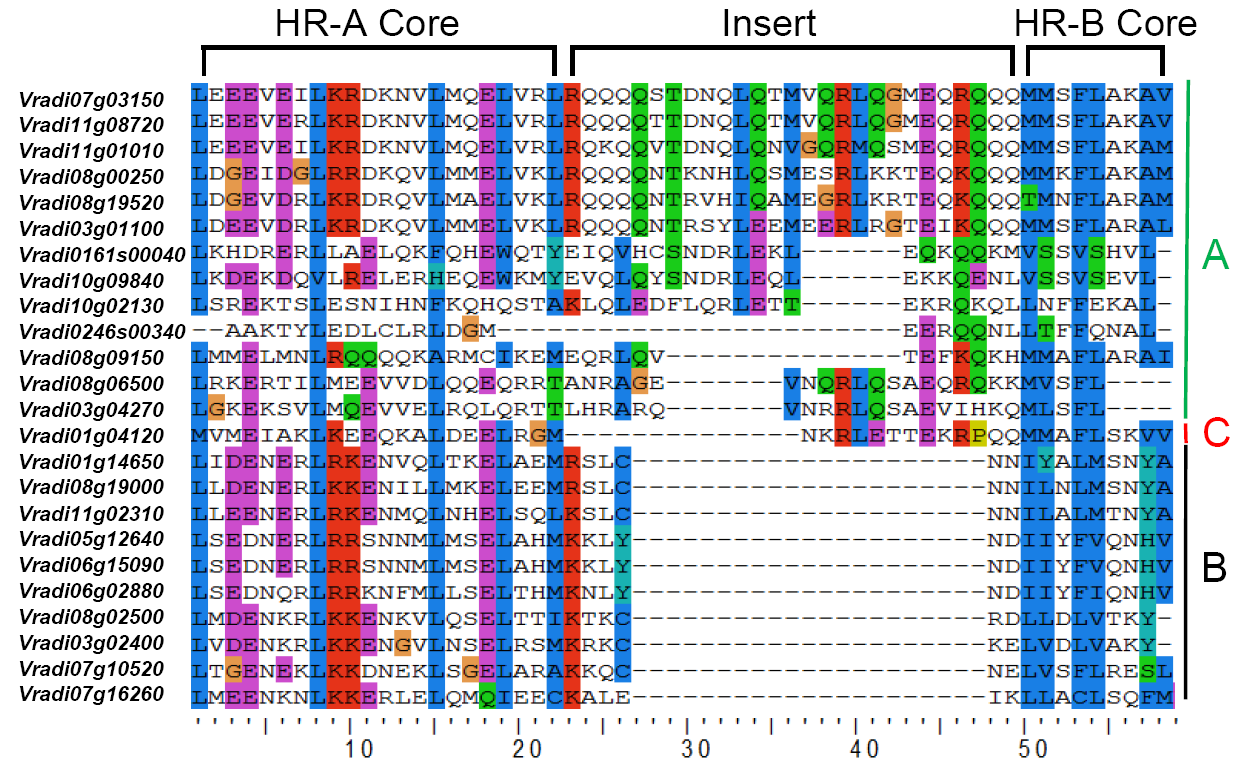


**Supplementary Figure 2. Alignment of VrHsf HR-A and HR-B regions**. The HR-A core, insert and HR-B core were highlighted.

**Supplementary Figure 3. Gene structure analysis of VrHsfA7b**. Conserved domains were shown in the diagram. The orange box indicates plant transposase domain, and the blue box means HSF-type DNA-binding domain. The numbers indicate the position of the amino acid of VrHsfA7b.
